# Household income and first psychiatric hospital admissions among children and adolescents

**DOI:** 10.1177/00207640251353675

**Published:** 2025-08-21

**Authors:** Veera Nieminen, Kimmo Suokas, Christian Hakulinen, Reija Autio, Sami Pirkola

**Affiliations:** 1 Faculty of Social Sciences, Health Sciences, Tampere University, Tampere, Finland; 2 Department of Psychology, University of Helsinki, Helsinki, Finland; 3 Finnish Institute for Health and Welfare, Helsinki, Finland; 4 Tampere University Central Hospital, Finland

**Keywords:** Mental disorders, hospital admissions, risk factors, gender differences, childhood, adolescence

## Abstract

**Background:**

Parental socioeconomic factors are associated with mental health outcomes already during childhood, but gender differences in these connections have rarely been studied.

**Aims:**

We explored the associations between household income and severe mental health disorders requiring psychiatric inpatient hospital care, with particular focus on gender differences.

**Methods:**

In this national register-based cohort study, we followed over 1.6 million children and adolescents born in Finland between 1991 and 2020 until first psychiatric hospital admission, moving away from parents, death, emigration from Finland or the end of 2020, whichever time came first. We calculated age- and gender-specific incidence rates (IRs) for first admissions. In order to evaluate gender differences and the magnitude of association between income and psychiatric hospital admission, we fitted multivariable Poisson regression models and calculated incidence rate ratios (IRRs) and 95% confidence intervals.

**Results:**

In total, 2.1% of the study population were admitted to psychiatric hospital for the first time within the specified period. Among girls in all income deciles, IRs distinctly peaked during adolescence. Among boys, IRs started to increase earlier, especially in the lowest income deciles, and there was no steep peak during adolescence in any income group. Lower household income was associated with higher risk for psychiatric hospital admission, and this association was steeper for boys (IRR 3.18 [2.87–3.53] than for girls (IRR 2.15 [1.97–2.35]) in the lowest compared to the highest income decile, after adjusting for potential confounders.

**Conclusion:**

Our results indicate that low income may play a more prominent role in severe mental disorders among boys, whereas adolescence emerges as a critical period for girls, regardless of their household income levels.

## Introduction

Mental disorders account for a high proportion of the disease burden in young people in all societies and have significant human and economic consequences (‘Global, Regional, and National Burden of 12 Mental Disorders in 204 Countries and Territories, 1990–2019’, GBD 2019 Mental Disorders Collaborators [2022]). The increased need for mental health treatment already during childhood and adolescence has not been met even in high-income countries, which is a major public health issue worldwide (Patel et al., 2007).

Socioeconomic status as a determinant for mental health has been established in low- and middle-income countries and in the Nordic countries, where the population generally has relatively equal access to health services (Kirkbride et al., 2024). The indicators of socioeconomic status reflect different aspects of the environment. Of these, income most directly measures the material resources available in everyday life, and can capture the dynamic changes (Galobardes, 2006). Previous research has rather consistently reported that growing up in low-income circumstances is associated with an increased risk of developing a mental disorder: children with parents from the lowest income percentile are estimated to be 3 to 4 times more likely to experience mental disorders compared to those in the highest income percentile (Kinge et al., 2021). The association between socioeconomic factors and mental disorders has been observed in all diagnostic groups, except in the case of eating disorders (Hakulinen et al., 2020; Kinge et al., 2021), and this link persists into adulthood (Evensen et al., 2021; Hakulinen et al., 2020).

In addition, gender differences in mental disorders are well-established. Girls are more frequently diagnosed with adolescent-onset anxiety and mood disorders (Campbell et al., 2021; Dalsgaard et al., 2020; Zahn-Waxler et al., 2008) and boys with childhood-onset conduct disorders and neuropsychiatric disorders (Zahn-Waxler et al., 2008). Due to these distinctions, gender-specific investigation of risk factors is well-founded. Evidence concerning gender differences in risk factors related to socioeconomic background is limited.

In this study, we investigated severe mental disorders among children and adolescents by examining psychiatric hospital admissions provided in cases of the most serious psychiatric symptoms and significant functional impairments or risk of causing harm to themselves or others (Kronström et al., 2023). Using nationwide registry data, we studied the incidence of psychiatric hospital admissions as well as disparities related to gender and the family’s socioeconomic status, measured by household income. We reported age- and gender specific incidence rates of admissions. We hypothesised that psychiatric hospital admissions would increase particularly during childhood in boys, and during adolescence in girls, and that the increase would be greater in the lowest income groups.

## Methods

**Study population and data sources:** This register-based cohort study included all individuals born in Finland from 1991 to 2020 (*n* = 1,643,611). Using population registers maintained by Statistics Finland, the study population was tracked from birth until they experienced one of the following events: their first psychiatric hospital admission; moving away from home; becoming non-dwelling; death; or the end of the study period (31st of December, 2020), whichever occurred first. Data were linked through record linkage. We included only those who had at least one Finnish-born parent, which covered 98.5% of the hospital admissions during the research period. This selection was made due to the incompleteness of income data for individuals who have migrated from another country.

**Household income measurements:** Household income was assessed using the OECD-modified Equivalence Scale (Förster & Marco, 2012), which calculated the equivalised disposable income for each household dwelling unit based on its size (Statistics Finland, 2024). The population was categorised into 10 income groups using deciles of the annual Finnish income distribution. Income deciles were based on income status on the last day of the previous year.

**Classification of mental disorders and assessment of psychiatric hospital admissions:** We identified mental disorders and first psychiatric hospital admissions by utilising the Finnish Hospital Discharge register, followed by the Care Register for Health Care, both maintained by the National Institute for Health and Welfare. We collected all first psychiatric inpatient treatment episodes and diagnoses for this study (Suokas, Gutvilig, et al., 2024). Since 1996, Finland has adopted the International Statistical Classification of Diseases and Related Health Problems, 10th Revision (ICD-10). We included all the ICD-10 diagnostic groups for mental, behavioural and neurodevelopmental disorders: mental disorders due to physiological conditions (F01–F09), substance-use disorders (F10–F19), psychotic disorders (F20–F29), mood disorders (F30–F39), anxiety disorders (F40–F48), behavioural syndromes (F50–59), personality disorders (F60–F69), intellectual disabilities (F70–F79), developmental disorders (F80–F89) and behavioural disorders (F90–F98).

**Gender:** This study utilised a binary gender categorisation. In population registers, sex is assigned at birth as male or female. Adults can change their recorded sex through self-declaration and be classified in the statistical gender category that aligns with their self-identified gender, either male or female. We used the term gender as it encompasses not only biological distinctions but also the social dimensions, including how individuals are perceived and treated as representatives of their gender in society.

**Cofactors:** For analyses adjusted for potential confounders, we included factors that could function as common causes for income level and for being admitted to a psychiatric hospital. Urbanicity was assessed with the seven-level urban–rural classification based on a nationwide grid of 250 × 250 m cells, in order to measure urbanicity for an individual’s place of residence (Finnish Environment Institute, 2013), visualised in article of Suokas, Kurkela, et al. (2024). To assess parental mental disorders, we detected whether either parent had a record of any mental disorder. These records were retrieved from psychiatric inpatient contacts since 1975, secondary outpatient care since 1998 and primary care since 2011 (Suokas, Gutvilig, et al., 2024). Parental education refers to the highest completed level: basic, upper secondary, short-cycle tertiary, lower degree tertiary or higher degree tertiary education. If information of the education was missing in the registers, it was coded as basic education. In addition, we included calendar year grouped as 1991-2000, 2001-2010 and 2011-2020, and parental age grouped as under 18, 19-30, 30-40 and over 40. All parental cofactors were collected separately for mothers and fathers.

**Statistical analyses**: We calculated age- and income-specific incidence rates (IRs) and 95% confidence intervals (CIs) for the first psychiatric hospital admission, considering any mental disorder, separately for boys and girls. Ages 0 to 5 were combined due to the modest number of hospital admissions, and ages 21 to 24 due to the decreasing amount of person-time at risk. We summed the number of admissions and the person-years at risk for the first admission across all combinations of the variables. Strata in the summed data were considered as the unit of analysis, and log person-time served as an offset term. In order to detect associations between outcome and exposures of interest, we used Poisson regression models with a robust sandwich variance estimator. First, we created minimally adjusted models including income and age. Then, to account for potential confounders, we fitted multivariable models including cofactors. We tested for interaction effects of income*age*gender and income*age and compared different models with Bayesian information criteria (BIC). The income*age interaction effect appeared to be significant, but the inclusion of this interaction had a negligible effect on the estimates. Additionally, we compared the different models with BIC and based on these comparisons and only minor changes in estimates, we chose separate models for boys and girls without the interaction term. We calculated income decile-specific incidence rate ratios (IRRs) and corresponding 95% CIs. For income, the reference group was the IRs of the highest decile. For age, we chose 12 for reference because of the equal number of admissions for both girls and boys. Statistical analyses utilised software R, version 4.3.1, and Stata, version 16.1.

## Results

In total, the study population included 1,643,611 children and adolescents (48.9% girls), contributing over 22.4 million person-years at risk. A total of 2.1% of the study population (34,387 persons) had a first psychiatric hospital admission during the research period. Girls accounted for 52.3% of the admissions, while boys accounted for 47.7%. At the ages of 15, 18, 20 and 24, 99.6%, 88.3%, 47.2% and 6.5% of the study population were still living with at least one of their parents, respectively. The characteristics of the study sample are described in Table 1.

**Table 1.** The characteristics of the study sample.

Characteristic	Admissions (%)		Person-time at risk, years (%)		Rate per 10,000	
	Boys	Girls	Boys	Girls	Boys	Girls
Household income decile						
1	3,380 (20.6)	2,806 (15.6)	1,099,133 (9.5)	1,052,737 (9.7)	30.75	26.66
2	2,315 (14.1)	2,422 (13.5)	1,037,589 (9.0)	976,926 (9.0)	22.31	24.79
3	2,156 (13.1)	2,362 (13.1)	1,182,341 (10.2)	1,107,654 (10.2)	18.24	21.32
4	1,851 (11.3)	2,072 (11.5)	1,271,131 (11.0)	1,187,071 (11.0)	14.56	17.45
5	1,583 (9.7)	1,906 (10.6)	1,313,166 (11.3)	1,224,878 (11.3)	12.05	15.56
6	1,372 (8.4)	1,639 (9.4)	1,280,635 (11.1)	1,191,736 (11.0)	10.71	14.21
7	1,124 (6.9)	1,454 (8.1)	1,210,545 (10.5)	1,121,893 (10.4)	9.29	12.96
8	916 (5.6)	1,192 (6.6)	1,077,328 (9.3)	995,927 (9.2)	8.50	11.97
9	696 (4.2)	958 (5.3)	885,985 (7.7)	817,424 (7.6)	7.86	11.72
10	495 (3.0)	715 (4.0)	738,889 (6.4)	691,728 (6.4)	6.70	10.34
Missing	122 (0.7)	84 (0.5)	446,068 (3.9)	426,449 (3.9)	2.73	1.97
Non-dwelling	392 (2.4)	321 (1.8)	41,946 (0.4)	39,900 (0.4)	93.45	80.45
Age						
0–5	318 (1.9)	131 (0.7)	4,598,541 (39.7)	4,401,160 (40.6)	0.69	0.30
6–10	6,069 (37.0)	1,680 (9.3)	3,080,375 (26.6)	2,959,610 (27.3)	19.70	5.68
11–15	6,023 (36.7)	10,487 (58.3)	2,334,124 (20.1)	2,247,739 (20.7)	25.80	46.66
16–20	3,188 (19.4)	5,248 (29.2)	1,224,967 (10.6)	1,058,072 (9.8)	26.03	49.60
21–25	804 (4.9)	439 (1.5)	346,765 (3.0)	167,742 (1.5)	23.19	26.17
Calendar year						
1991–2000	354 (2.2)	114 (0.6)	1,583,317 (13.7)	1,520,932 (14.0)	2.24	0.75
2001–2010	6,400 (39.0)	5,111 (28.4)	4,361,053 (37.6)	4,173,281 (38.5)	14.68	12.25
2011–2020	9,648 (58.8)	12,760 (70.9)	5,640,400 (48.7)	5,140,110 (47.4)	17.11	24.82
Urbanicity						
Inner Urban	3,951 (24.1)	4,270 (23.7)	2,501,979 (21.6)	2,371,014 (21.9)	15.80	18.01
Outer Urban	5,025 (30.6)	5,252 (29.2)	3,374,369 (29.1)	3,167,245 (29.2)	14.90	16.58
Peri-urban	1,934 (11.8)	2,349 (13.1)	1,576,215 (13.6)	1,462,121 (13.5)	12.27	16.07
Rural Local Center	1,027 (6.3)	1,191 (6.6)	660,648 (5.7)	622,934 (5.7)	15.55	19.12
Rural Close to Urban	1,340 (8.2)	1,516 (8.4)	988,752 (8.5)	912,090 (8.4)	13.56	16.62
Rural Heartland	1,908 (11.6)	2,107 (11.7)	1,389,220 (12.0)	1,275,033 (11.8)	13.73	16.53
Sparsely Populated Rural	884 (5.4)	1,021 (5.7)	625,255 (5.4)	574,850 (5.3)	14.14	17.76
Missing	333 (2.0)	279 (1.6)	468,334 (4.0)	449,037 (4.1)	7.11	6.21
Mother’s age at birth						
Under 18	363 (2.2)	296 (1.6)	80,299 (0.7)	73,701 (0.7)	45.21	40.16
18–30	9,879 (60.2)	10,420 (57.9)	6,296,769 (54.4)	5,875,654 (54.2)	15.69	17.73
30–40	5,736 (35.0)	6,727 (37.4)	4,893,017 (42.2)	4,590,099 (42.4)	11.72	14.66
Over 40	412 (2.5)	532 (3.0)	298,573 (2.6)	279,400 (2.6)	13.80	19.04
Missing	12 (0.1)	10 (0.1)	16,112 (0.1)	15,469 (0.1)	7.44	6.46
Mother’s mental disorder						
Yes	7,427 (45.3)	8,086 (44.9)	2,048,452 (17.7)	1,889,045 (17.4)	36.26	42.71
No	8,975 (54.7)	9,917 (55.1)	9,536,319 (82.3)	8,945,279 (82.6)	9.41	11.09
Mother’s education						
Basic	4,695 (28.6)	5,245 (29.2)	2,221,290 (19.2)	2,046,268 (18.9)	21.14	25.63
Upper secondary	7,025 (42.8)	7,018 (39.0)	4,341,550 (37.5)	4,075,660 (37.6)	16.18	17.22
Short-cycle tertiary	2,133 (13.0)	2,630 (14.6)	2,069,134 (17.9)	1,940,361 (17.9)	10.31	13.55
Lower degree tertiary	1,305 (8.0)	1,440 (8.0)	1,374,984 (11.9)	1,298,287 (12.0)	9.49	11.09
Higher degree tertiary	1,244 (7.6)	1,652 (9.2)	1,577,813 (13.6)	1,473,747 (13.6)	7.88	11.21
Father’s age at birth						
Under 18	105 (0.6)	83 (0.5)	20,087 (0.2)	19,315 (0.2)	52.27	42.97
18–30	7,400 (45.1)	7,726 (43.0)	4,612,161 (39.8)	4,305,592 (39.7)	16.04	17.94
30–40	6,739 (41.1)	7,991 (44.4)	5,715,681 (49.3)	5,345,880 (49.3)	11.79	14.95
Over 40	1,659 (10.1)	1,757 (9.8)	1,062,296 (9.2)	997,155 (9.2)	15.62	17.62
Missing	499 (3.0)	428 (2.4)	174,546 (1.5)	166,382 (1.5)	28.59	25.72
Father’s mental disorder						
Yes	5,344 (32.6)	5,470 (30.4)	1,555,869 (13.4)	1,441,606 (13.3)	34.35	37.94
No	11,058 (67.4)	12,515 (69.6)	10,028,901 (86.6)	9,392,717 (86.7)	11.03	13.32
Father’s education						
Basic	6,444 (39.3)	6,975 (38.8)	2,982,801 (25.7)	2,781,713 (25.7)	21.60	25.07
Upper secondary	6,705 (40.9)	6,917 (38.5)	4,877,085 (42.1)	4,557,845 (42.1)	13.75	15.18
Short-cycle tertiary	1,291 (7.9)	1,510 (8.4)	1,265,958 (10.9)	1,173,641 (10.8)	10.20	12.87
Lower degree tertiary	966 (5.9)	1,169 (6.5)	1,114,092 (9.6)	1,053,085 (9.7)	8.67	11.10
Higher degree tertiary	996 (6.1)	1,414 (7.9)	1,344,835 (11.6)	1,268,041 (11.7)	7.41	11.15

**Figure 1.**
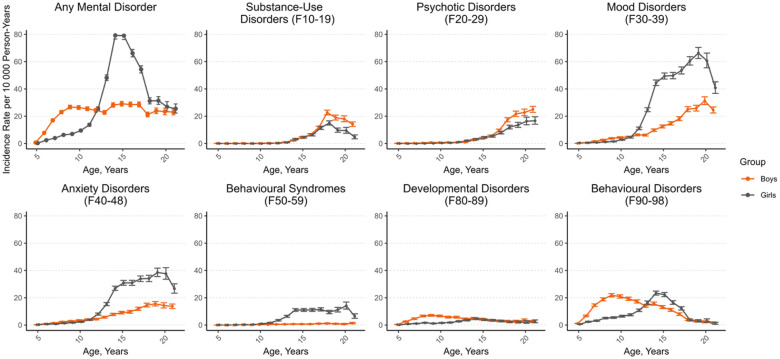
Incidence rate of first psychiatric hospital admissions by age, gender, and diagnostic group. Data markers denote the incidence rate (IR) per 10,000 person-years; error bars, 95% CIs. Diagnostic groups are based on the ICD-10 sub-chapter categories of mental and behavioural disorders.

Gender-specific incidence rates of first psychiatric hospital admissions due to mental disorder during childhood and adolescence are shown in Figure 1. Among boys, the incidence rate increased during childhood, after which it remained at a quite steady level. Among girls, the incidence rate increased rapidly from age 12, peaking at the age of 15 years and settling near the same level as boys at the age of 20 years. Of those who were admitted, 67.6% had one, 27.1% two and 5.3% three or more diagnoses. Boys had a higher rate of hospital admissions due to substance use and psychotic disorders from age 15 onwards. Behavioural disorders were more common among boys at ages 6 to 12 and among girls at ages 13 to 17. Girls had a higher rate of mood and anxiety disorders and behavioural syndromes from age 13 onwards. Mental disorders due to physiological conditions, personality disorders and intellectual disabilities accounted for only a minor proportion (0.1%, 1.1% and 1.1%) of the admissions and are therefore not presented in the figure.

**Figure 2.**
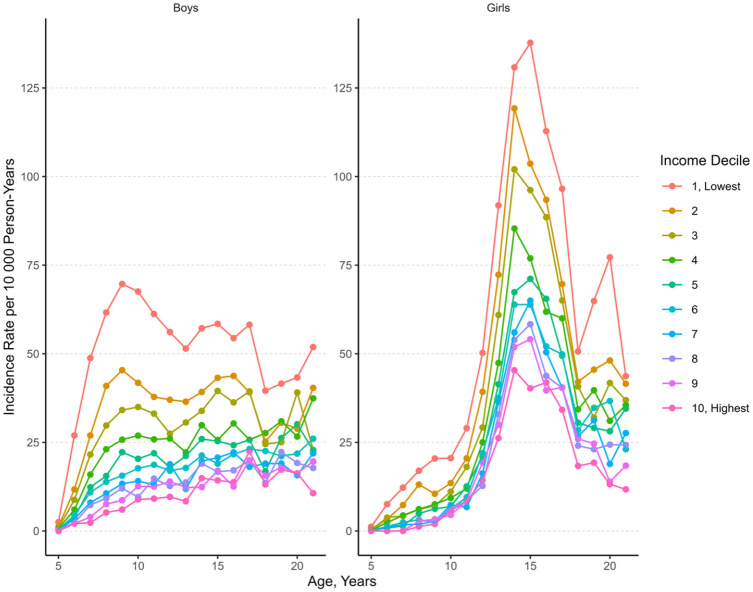
Incidence rate of first psychiatric hospital admissions by age, gender and household income. Data markers denote the incidence rate (IR) per 10,000 person-years. Income decile refers to equivalised disposable income deciles of a household, with 1 indicating the lowest income decile and 10, the highest.

Age- and income-specific incidence rates of psychiatric hospital admissions due to any mental disorder diagnosis for boys and girls are presented in Figure 2. The incidence rate was highest in the lowest income decile group after the age of 5 throughout childhood and adolescence, particularly in boys. In girls, a peak in the incidence rate emerged around the age of 15 years in all income deciles.

**Figure 3.**
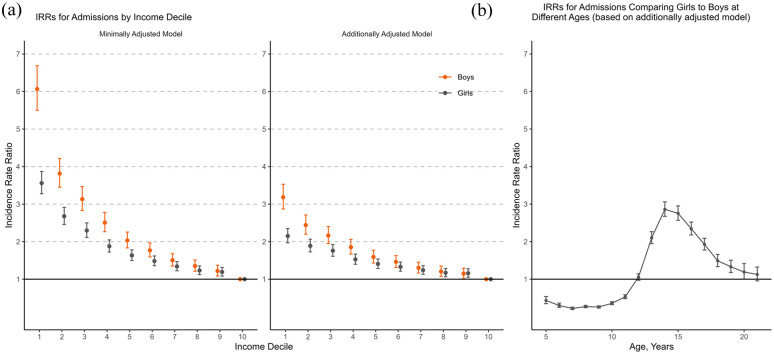
(a) Incidence rate ratios (IRRs) for first psychiatric hospital admissions by income decile, reported separately for boys and girls. (b) Incidence rate ratios (IRRs) for first psychiatric hospital admissions comparing girls to boys at different ages. Incidence rate ratios (IRRs) calculated using Poisson regression. The minimally adjusted model includes income decile and age as exposures. The additionally adjusted model includes also calendar year, urbanicity and parental age, education, and mental disorder. Income decile refers to equivalised disposable income deciles of a household, with 1 indicating the lowest income decile and 10 the highest (reference group); error bars, 95% CIs.

The results of Poisson regression models are presented in Table 2. Gender comparisons for income-related associations are shown in Figure 3(a), and for age-specific incidence rate ratios in Figure 3(b). In a minimally adjusted model including income decile and age as exposures, low income was associated with higher IRR for psychiatric hospital admission (in the lowest income decile compared to the highest IRR = 6.07, 95% CI [5.50, 6.69] among boys and IRR = 3.56, 95% CI [3.27, 3.87] among girls). In a model adjusted for potential confounders including urbanicity, calendar year and parental age, education and mental disorder, the association between income and risk for psychiatric hospital admission was attenuated but remained (among boys IRR = 3.18, 95% CI [2.87, 3.53] in the lowest income decile compared to the highest and among girls IRR = 2.15, 95% CI [1.97, 2.35] in the lowest income decile compared to the highest). Furthermore, living in an urban area, parental mental disorder, mother’s low education, mother’s younger age and father’s advanced age were associated with increased risk for psychiatric hospital admission for both boys and girls. Mother’s advanced age increased the risk only for girls, and father’s lower education and younger age only for boys.

**Table 2.** Risk factors for psychiatric hospital admissions.

Characteristic	Boys				Girls			
	Minimally adjusted		Additionally adjusted		Minimally adjusted		Additionally adjusted	
	IRR	95% CI	IRR	95% CI	IRR	95% CI	IRR	95% CI
Household income decile								
1	6.07	5.50, 6.69	3.18	2.87, 3.53	3.56	3.27, 3.87	2.15	1.97, 2.35
2	3.81	3.45, 4.22	2.44	2.20, 2.71	2.68	2.46, 2.91	1.89	1.73, 2.06
3	3.13	2.83, 3.46	2.16	1.95, 2.40	2.30	2.11, 2.50	1.76	1.61, 1.92
4	2.51	2.26, 2.78	1.85	1.67, 2.06	1.88	1.72, 2.05	1.53	1.40, 1.67
5	2.03	1.83, 2.26	1.59	1.43, 1.77	1.63	1.50, 1.78	1.41	1.29, 1.54
6	1.77	1.59, 1.97	1.46	1.31, 1.63	1.48	1.36, 1.62	1.33	1.21, 1.45
7	1.50	1.35, 1.68	1.30	1.17, 1.46	1.34	1.22, 1.47	1.24	1.13, 1.36
8	1.35	1.21, 1.51	1.21	1.08, 1.35	1.23	1.12, 1.35	1.17	1.06, 1.29
9	1.22	1.08, 1.37	1.15	1.02, 1.29	1.19	1.08, 1.31	1.16	1.05, 1.28
10 (reference)	1		1		1		1	
Age								
0–5	0.03	0.02, 0.03	0.03	0.03, 0.03	0.01	0.01, 0.01	0.01	0.01, 0.02
6	0.31	0.27, 0.34	0.32	0.29, 0.36	0.09	0.08, 0.11	0.09	0.08, 0.11
7	0.68	0.62, 0.74	0.70	0.64, 0.76	0.15	0.13, 0.17	0.15	0.13, 0.17
8	0.92	0.85, 1.00	0.94	0.87, 1.02	0.24	0.21, 0.27	0.25	0.22, 0.28
9	1.07	0.99, 1.16	1.08	1.00, 1.17	0.26	0.23, 0.30	0.27	0.24, 0.31
10	1.07	0.99, 1.16	1.08	1.00, 1.17	0.37	0.33, 0.41	0.37	0.34, 0.42
11	1.05	0.97, 1.14	1.05	0.97, 1.14	0.54	0.48, 0.59	0.54	0.49, 0.59
12 (reference)	1		1		1		1	
13	0.96	0.88, 1.04	0.96	0.88, 1.05	1.92	1.78, 2.07	1.90	1.76, 2.04
14	1.16	1.07, 1.26	1.17	1.08, 1.27	3.14	2.92, 3.37	3.10	2.90, 3.32
15	1.18	1.09, 1.28	1.20	1.10, 1.30	3.10	2.88, 3.33	3.02	2.82, 3.24
16	1.18	1.09, 1.29	1.21	1.11, 1.32	2.63	2.45, 2.84	2.54	2.36, 2.73
17	1.23	1.13, 1.35	1.25	1.14, 1.36	2.22	2.05, 2.40	2.10	1.95, 2.27
18	0.97	0.87, 1.07	1.01	0.91, 1.11	1.34	1.22, 1.47	1.26	1.15, 1.38
19	1.17	1.05, 1.30	1.24	1.11, 1.37	1.43	1.28, 1.59	1.32	1.18, 1.47
20	1.20	1.06, 1.35	1.25	1.11, 1.41	1.28	1.11, 1.49	1.16	1.00, 1.34
21–24	1.27	1.13, 1.41	1.26	1.13, 1.41	1.25	1.08, 1.45	1.10	0.95, 1.27
Calendar year								
1991–2000 (reference)			1				1	
2001–2010			1.27	1.13, 1.43			0.95	0.77, 1.18
2011–2020			0.93	0.82, 1.04			1.03	0.83, 1.27
Urbanicity								
Inner urban			1.37	1.27, 1.48			1.18	1.10, 1.27
Outer urban			1.34	1.24, 1.44			1.10	1.02, 1.18
Peri-urban			1.10	1.01, 1.19			1.05	0.97, 1.13
Rural local centre			1.21	1.11, 1.33			1.11	1.02, 1.21
Rural close to urban			1.09	1.00, 1.19			1.02	0.94, 1.10
Rural heartland			1.06	0.97, 1.14			0.98	0.91, 1.06
Sparsely populated rural (reference)			1				1	
Mother’s age at birth								
Under 18			1.57	1.37, 1.79			1.38	1.19, 1.59
18–30			1.07	1.03, 1.11			1.04	1.00, 1.08
30–40 (reference)			1				1	
Over 40			0.99	0.89, 1.10			1.19	1.09, 1.31
Mother’s mental disorder								
Yes			2.63	2.53, 2.72			2.51	2.43, 2.59
No (reference)			1				1	
Mother’s education								
Basic (reference)			1				1	
Upper secondary			1.09	1.04, 1.14			0.99	0.94, 1.03
Short-cycle tertiary			0.89	0.84, 0.95			0.86	0.82, 0.91
Lower degree tertiary			0.97	0.90, 1.04			0.89	0.84, 0.96
Higher degree tertiary			0.98	0.91, 1.06			0.93	0.87, 1.00
Father’s age at birth								
Under 18			1.47	1.17, 1.85			1.30	1.00, 1.68
18–30			1.06	1.02, 1.11			1.01	0.98, 1.05
30–40 (reference)			1				1	
Over 40			1.18	1.12, 1.26			1.06	1.01, 1.13
Father’s mental disorder								
Yes			1.80	1.74, 1.87			1.63	1.58, 1.69
No (reference)			1				1	
Father’s education								
Basic (reference)			1				1	
Upper secondary			0.90	0.86, 0.94			0.93	0.89, 0.96
Short-cycle tertiary			0.78	0.73, 0.84			0.83	0.78, 0.88
Lower degree tertiary			0.82	0.76, 0.89			0.93	0.87, 1.00
Higher degree tertiary			0.76	0.71, 0.83			0.94	0.88, 1.01

Results of Poisson regression models, reported separately for boys and girls; incidence rate ratios (IRRs) and 95% CIs. The minimally adjusted model includes income decile and age as exposures. The additionally adjusted model includes all variables presented in the table.

## Discussion

Our findings suggest that there are gender differences in psychiatric hospital admissions during childhood and adolescence. Low income appears to play a more prominent role among boys, whereas adolescence emerges as a critical period for girls, regardless of their household income levels. To the best of our knowledge, this study is the first to highlight the gender dependence of the risk factors for psychiatric hospital admissions.

The incidence of hospital admissions among boys began to increase during childhood, after which their levels remained relatively stable. Conversely, among girls, the incidence of hospital admissions peaked rapidly during adolescence. At age 15, girls had over 2.5 times the number of first psychiatric hospital admissions in comparison to boys. Previously, gender-specific frequencies of mental disorders have often been studied in selected diagnostic groups. Among studies that have examined the full diagnostic spectrum, Swedish (Yang et al., 2024) and Danish (Dalsgaard et al., 2020) register-based studies reported a higher incidence of any mental disorder during adolescence among girls. In addition, a British study examining psychiatric hospitalisations during the years 1998-2004 reported higher rates among girls starting from the age of 13 (James et al., 2010). Our results are in line with this finding, indicating a pronounced increase in adolescent girls’ psychiatric hospital admissions from age 12. Register-based studies (Dalsgaard et al., 2020; Yang et al., 2024) have found that the incidence of any mental disorder increased during adolescence also among boys. However, in our study, the incidence of psychiatric hospital admissions remained at a quite steady level among boys. This raises the question of whether girls are more susceptible to the most severe mental disorders during adolescence.

The gender- and age distribution of diagnoses accounting for psychiatric hospital admissions is consistent with previous studies. Boys displayed a preponderance of behavioural disorders at ages 6 to 12, whereas girls showed a preponderance at ages 13 to 17. Mood and anxiety disorders and behavioural syndromes increased and became remarkably more common in girls from age 13. These findings are consistent with those of previous studies, which have identified male preponderance in emotional disorders before the age of 12 years, followed by a shift towards female preponderance (Wesselhoeft et al., 2015). Moreover, it has been proposed that girls might experience a delayed detection of mental disorders, particularly neurodevelopmental disorders (Dalsgaard et al., 2020). The existing literature suggests that boys may have a general pre-pubertal vulnerability to a broader spectrum of mental disorders (Dalsgaard et al., 2020; Wesselhoeft et al., 2015), and our findings align with this hypothesis.

The association between income and risk for psychiatric admission remained after adjusting for urbanicity, calendar year, parental mental disorder, parental education and parental age. Gender did not function as an effect modifier and the association between household income and risk for psychiatric hospital admission was observed in both genders, although there were differences in magnitude. After adjusting the models for potential confounders, boys in income deciles 1-3 had higher IRRs for hospital admissions than girls. To the best of our knowledge, results indicating boys in low-income groups to be more liable to hospital-treated mental disorder than girls have not been reported earlier. An income gradient for children under 18 years has been established in psychiatric hospital admissions (Suokas et al., 2020) as well as for mental disorders treated in all health services (Kinge et al., 2021). However, in the study on hospital admissions, the models were not adjusted for parental characteristics. Previous findings on gender differences in these associations are mixed. A systematic review of socioeconomic inequalities and mental health during childhood and adolescence summarised that there were no substantial gender differences (Reiss, 2013). It has been reported that socioeconomic inequalities in childhood mental disorders in general would be stronger for boys, although in the cited study income was only partly associated with an increased risk for mental disorders after adjusting for control variables (Vaalavuo et al., 2022). In addition, one study on mental disorders across the whole lifespan reported that the incidence rate differences between the sexes were greater among individuals with lower socioeconomic status (Yang et al., 2024).

There may be several explanations for results concerning gender differences in psychiatric hospital care. The pronounced incidence peak among girls during adolescence may be attributed to various gendered factors, including hormonal changes during puberty and early maturation (Zahn-Waxler et al., 2008), exposure to sexual discrimination (Klonoff et al., 2000), and pressures related to school performance (Wiklund et al., 2012) and physical appearance (Grabe et al., 2007). Furthermore, gender disparities in overall mental health tend to be more prevalent in wealthier and more gender-equal countries such as the Nordic countries, which has been attributed to the dual requirements stemming from both modern and traditional gender roles (Campbell et al., 2021; Yu, 2018).

Our results raise the question of whether boys are more vulnerable to socioeconomic adversity experienced during childhood, and whether higher household income may exert a more pronounced protective effect on boys. Some research on health economics posits that adverse social environments during childhood potentially lead to poorer outcomes in disciplinary problems and educational achievement (Autor et al., 2019), and adult employment among males (Chetty et al., 2016). Some evidence also suggests that socioeconomic risk factors are more notably pronounced in externalising disorders, which have a higher prevalence among boys during childhood (Peverill et al., 2021; Reiss, 2013).

The causal relationship between socioeconomic status and mental health is debated. Economic hardship can act as a stressor impacting mental health, while mental disorders may limit socioeconomic achievement (Mossakowski, 2014; Reiss, 2013). Childhood poverty has been established to be linked with dysregulated stress responses (Evans & Kim, 2007). Economic strain may affect child development through stress, interparental conflicts and parenting behaviours, even in welfare states with social security (Conger & Ge, 1994; Solantaus et al., 2004). Economic disadvantage may affect access to early interventions, with parents having a significant role in seeking for and participating in treatment. In Finland, mental health services are free for children, but the visits may still require everyday arrangements and resources from the parents, and place burdens on families. Children from low-income families may have less access to high-quality mental health services (Hodgkinson et al., 2017). Studies on adults suggest that low-income individuals face more barriers to care, although the evidence on parental socioeconomic factors and help-seeking for children is contradictory (Ryan et al., 2015; Sareen et al., 2007; Zwaanswijk et al., 2003).

This study has several strengths, namely that the results are population-based and free of health-related selection and non-participation biases. It also encounters some important limitations. First, our measures of financial resources may not describe the entirety of a family’s economic reality. We used equivalised household income as a measure and did not consider other forms of wealth and property, which are not captured with the national statistics. Moreover, lack of social support may be an important explanatory factor, but it is not available in the data. Nevertheless, household income probably serves as a sensitive indicator of the available material resources that parents can invest in their children (Galobardes, 2006). Second, our examination focused solely on one Nordic country, which may impact the applicability of the results. Moreover, the presence of extensive public health care and numerous free services in Finland may not reflect the health care realities of other countries. Third, the study employed a binary variable of allocated gender in population registers and did not differentiate the subjects by their experienced gender or identify gender minorities. It would be important to consider this issue in future research, since for example, transgender identity is associated with suicidal ideation and suicide attempts among adolescents and young adults (Erlangsen et al., 2023), and presumably therefore with a higher risk for psychiatric hospital care. Lastly, it is worth acknowledging that even high-quality register data are subject to certain constraints, including some uncertainty concerning the validity of the diagnoses (Sund, 2012). However, this study utilised psychiatric hospital admissions which signal significant concerns for mental well-being. Additionally, the coverage of national registry data is not as comprehensive for the migrant populations. In the context of income, it would nevertheless be important to consider, for example, precarious employment within immigrant populations, the key dimensions of which are not adequately captured in registries (Gauffin, 2022). This study excluded foreign-born individuals and those whose parents were both foreign-born, so the results are not generalisable to individuals whose parents are not of Finnish origin. In Finland, immigrant populations constitute a heterogeneous group with varying levels of pre-migration stressors and post-migration adversities (OECD, 2024). It would be important to include and focus on migrant populations in future research, given their unique psychosocial and mental health challenges, particularly in the case of individuals migrating from conflict-affected areas (Hazer & Gredebäck, 2023).

In conclusion, our findings on psychiatric hospital care indicate that girls exhibit vulnerability during adolescence irrespectively of their household income. In boys, household income plays a relatively more important role. These results suggest that low income as a risk factor for hospital-treated mental health disorder is gendered from an early age and highlights the need to consider a gender-informed approach in treatment and prevention. Removing barriers and ensuring equitable access to preventive mental health care for children and adolescents from low-income families might help in mitigating the onset of acute psychiatric conditions, especially in boys. The age-related vulnerability observed in adolescent girls underscores the importance of better recognition and management of this issue in mental health service strategies. Efforts to address mental disorders should be extended to broader societal and political reforms aimed at preventing their emergence.
